# Clinicopathologic correlations of renal biopsy findings from northeast China: A 10-year retrospective study

**DOI:** 10.1097/MD.0000000000015880

**Published:** 2019-06-07

**Authors:** Sensen Su, Jinyu Yu, Yue Wang, Yu Wang, Jia Li, Zhonggao Xu

**Affiliations:** Department of Nephrology, The First Hospital of Jilin University, Changchun, China.

**Keywords:** clinical indication, IgA nephropathy, membranous nephropathy, nephrotic syndrome, renal biopsy

## Abstract

Renal biopsy is the cornerstone of diagnostic approaches in nephrology, as they provide invaluable diagnostic information. In this study, we analyzed and reported renal biopsy results from northeast China from the past 10 years to describe the epidemiological trend.

We analyzed clinical features, indications, and histological diagnoses of renal biopsies collected between January 1, 2007, and December 31, 2016.

There were 2725 identified cases (with a mean age of 41.24 ± 15.18 years, 55% male) during the study period. The main clinical indication was nephrotic syndrome (59.9%). Membranous nephropathy (29.1%) was the most common pathological finding in the entire study population, followed by IgA nephropathy (23.4%), minimal change disease (12.7%), and mesangio-proliferative glomerulonephritis (7.4%).

We divided the study period into 2 subperiods: 2007 to 2011 (period 1) and 2012 to 2016 (period 2). Membranous nephropathy and minimal change disease were more frequent in period 2 than in period 1. Conversely, IgAN and non-IgA mesangio-proliferative glomerulonephritis were less frequent in period 2 than in period 1. Cases of Henöch–Schönlein purpura nephritis and lupus nephritis were observed less over time, while cases of nephroangiosclerosis increased significantly over time. Finally, there was a significant increase in the number of tubulointerstitial diseases observed over time, while there was a significant decrease in glomerulosclerosis and unclassified findings over time.

Membranous nephropathy was the most common pathological finding from renal biopsy and the prevalence has increased significantly in recent years in northeast China.

## 1 Introduction

Percutaneous renal biopsy is the cornerstone of diagnostic approaches in nephrology and provides important therapeutic and prognostic information for practicing nephrologists. The spectrum of renal biopsy findings differs depending on the geographical area, ethnicity, and environmental and socioeconomical factors. IgA nephropathy (IgAN) is the predominant pathological result from renal biopsy in several European countries,^[1,2]^ Australia,^[3]^ and most countries in Asia.^[4–6]^ Conversely, in the United States^[7,8]^ and Brazil,^[9]^ focal segmental glomerulosclerosis (FSGS) is the predominant pathological result. While membranoproliferative glomerulonephritis (MPGN) is the most common pathology in South Africa,^[10]^ and membranous nephropathy (MN) is the most common pathology from renal biopsy in Spain.^[11]^

The spectrum of renal biopsy findings also changes over time. For instance, the prevalence of MN has increased progressively in some regions,^[12]^ and IgAN has become the leading observed pathology in young Americans.^[13]^ The understanding of local epidemiology and the prevalence of different renal biopsy findings over time can help nephrologists gain a clearer picture of the origin of renal disease. This insight can, in turn, improve the diagnosis, prognosis prediction, and therapeutic planning in these patients. Thus, it is of great importance to examine the changing epidemiology of renal biopsy findings in any given areas. There are 9 countries^[2,4,9,11,14–18]^ that regularly update their local data every few years through their national renal biopsy registry. However, China has not yet established a nationwide registry, although there are anecdotal reports from middle^[19,20]^ and southern China,^[21]^ whose results differ from each other. Reported data from northern China are scarce. Therefore, we performed a retrospective study that described the epidemiology of renal biopsy findings from northeast China, as well as their changing prevalence over the past 10 years.

## 2 Materials and methods

This retrospective study was conducted between January 1, 2007, and December 31, 2016, in the largest nephrology center in Changchun, located in northeast China. The study protocol was approved by the Ethics Committee of the First Hospital of Jilin University. We collected demographic data from all participants undergoing renal biopsy, including the age at the time of renal biopsy (≥14 years of age), gender, the indications for renal biopsy, and the histological diagnosis. The specimens were stained with hematoxylin and eosin (H&E), periodic acid Schiff, Masson trichrome, and Congo-Red stain if necessary, and analyzed through light microscopy. Immunohistochemistry staining included IgG, IgM, IgA, C3, C4, and fibrinogen, and electron microscopy was used whenever necessary. All the biopsy slides were reviewed by the same pathologist.

Clinical indications were divided into 5 categories: nephrotic syndrome, defined as the presence of heavy proteinuria (24-hour urine protein ≥3.5 g) accompanied by serum albumin <30 g/L; nephritic syndrome, defined as the presence of 24-hour urine protein between 1.5 and 3.5 g with hematuria, with or without high blood pressure and edema; urinary abnormalities (UABs), defined as the presence of 24-hour urine protein <1.5 g, or hematuria without high blood pressure or edema; acute kidney injury (AKI) defined as the presence of declining estimated glomerular filtration rate (eGFR) within 3 months; and chronic kidney disease (CKD), defined as the presence of a decreased eGFR for ≥3 months.

Histopathological findings were divided into 6 major categories: primary glomerular disease (PGD); secondary glomerular disease (SGD); tubulointerstitial disease (TID); hereditary and congenital renal disease (HCRD); glomerulosclerosis; and unclassified. PGD was further subdivided into 8 categories, including IgAN, mesangio-proliferative glomerulonephritis (MsPGN), MN, minimal change disease (MCD), FSGS, MPGN, endocapillary glomerulonephritis (EnCGN), and crescentic glomerulonephritis (CREGN). SGD was subdivided into 5 categories, including systemic disease-related renal impairments such as lupus nephritis (LN) and Henöch–Schönlein purpura nephritis (HSPN); infection-associated renal impairments such as hepatitis B virus associated glomerulonephritis (HBV-GN), hepatitis C virus associated glomerulonephritis, and epidemic hemorrhagic fever-related renal damage; renal diseases related to vessels included nephroangiosclerosis (NeSS), systemic vasculitis, and thrombotic microangiopathy; metabolic disease associated renal damage such as diabetic nephropathy (DN), amyloidosis, cast nephropathy, monoclonal immunoglobulin deposition nephropathy, and obesity-related glomerulomegaly; and others such as pregnancy-related nephropathy. In this category, we mainly focused on HSPN, LN, HBV-GN, DN, NeSS, and amyloidosis. TID consisted of acute interstitial nephritis, acute tubular necrosis, and acute tubulointerstitial nephropathy. Hereditary and congenital kidney disease included thin basement membrane nephropathy, Fabry disease, and Alport syndrome, while other diagnoses included glomerulosclerosis and unclassified lesions, which were difficult to diagnose according to the modified WHO classification (1995).

## 3 Data analysis

Each patient who underwent renal biopsy in this center was given a questionnaire to assess the relevant demographic features and clinical indications for renal biopsy, and the questionnaire was answered by his/her doctor. These data were recorded in a system that contained the histopathological diagnoses of the patients and was digitally stored. The data were reformatted into a standard Microsoft excel file and were analyzed using the SPSS statistical package version 18.0 (IBM Corp., Chicago, IL). The frequencies of cases were expressed as percentages. χ^2^ test was used to determine the differences in disease frequencies according to gender, age group, and study periods in which the biopsy was performed. A *P* value of less than .05 was considered statistically significant.

## 4 Results

A total of 2725 cases were identified after excluding 626 recipients who underwent allograft biopsies, 363 young recipients (younger than 14 years of age), and 33 recipients with incomplete data. The mean age at the time of renal biopsy was 41.24 ± 15.18 years, with a large majority of the population falling within the age category of 20 to 64 years (Table 1). Male patients comprised the majority of the overall population (Table 2). The spectrum of histological findings differed according to gender (*P* < .05) (Table 2). PGD was more common in males than in females (*P* < .01), while SGD exhibited the opposite trend (*P* < .01). Specifically, patients with MN and MCD were more likely to be male (*P* < .01), while other PGD types did not show significant gender-related differences. For SGD, female patients were more likely to have LN (*P* < .01), while male patients comprised a majority of those with HBV-GN (*P* < .01), NeSS (*P* < .01), and amyloidosis (*P* < .05). No gender-related differences were observed in patients with HSPN, DN, TID, HCRD, glomerulosclerosis, and the unclassified group.

**Table 1:**
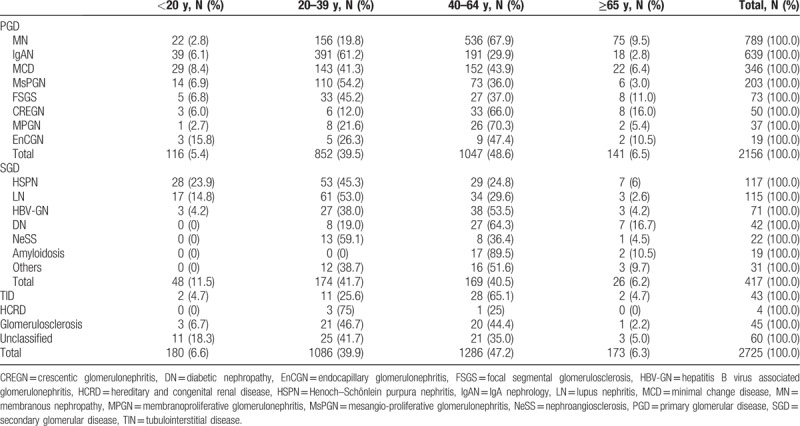
The distribution of pathological findings of renal biopsy based on different age groups.

**Table 2:**
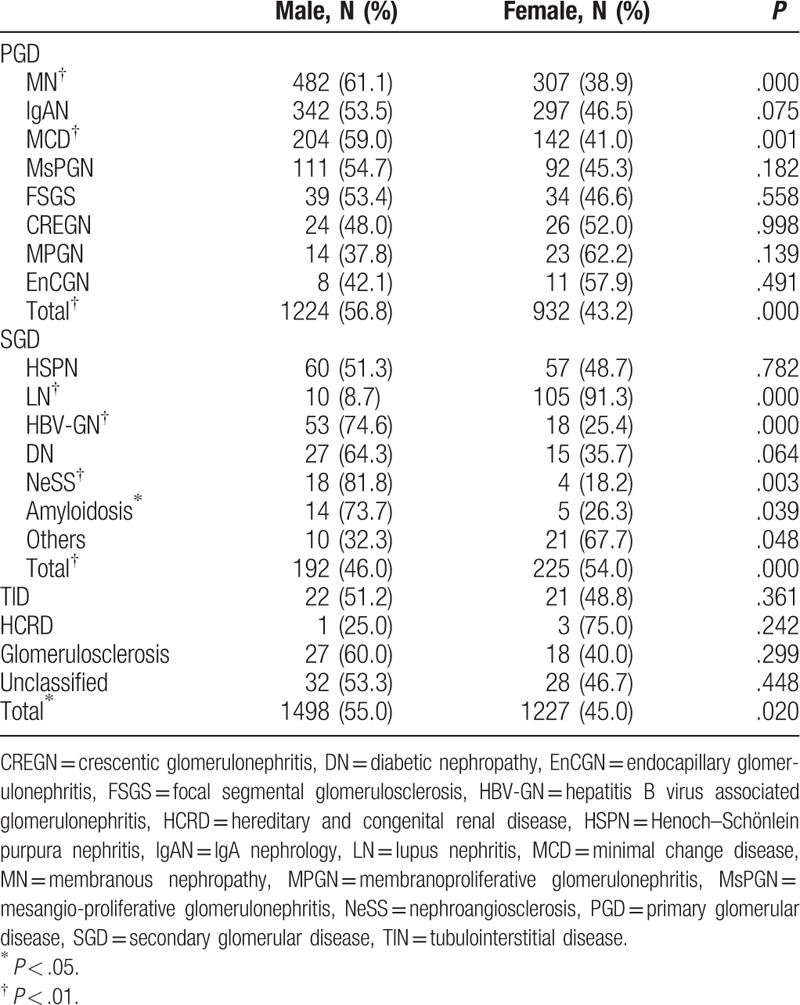
The distribution of pathological findings of renal biopsy based on gender.

The main clinical indication for renal biopsy was nephrotic syndrome, followed by nephritic syndrome, UAB, AKI, and CKD (Table 3). MN was the most common pathological finding in the whole study population, followed by IgAN, MCD, and MsPGN. HSPN and LN were the most common SGDs, followed by HBV-GN (N = 71, 2.6%), DN, NeSS, and amyloidosis (Tables 1 and 3). HBV-GN contains 2 main pathological types, including 69/71 MN and 2/71 mesangio-proliferative-GN.

**Table 3:**
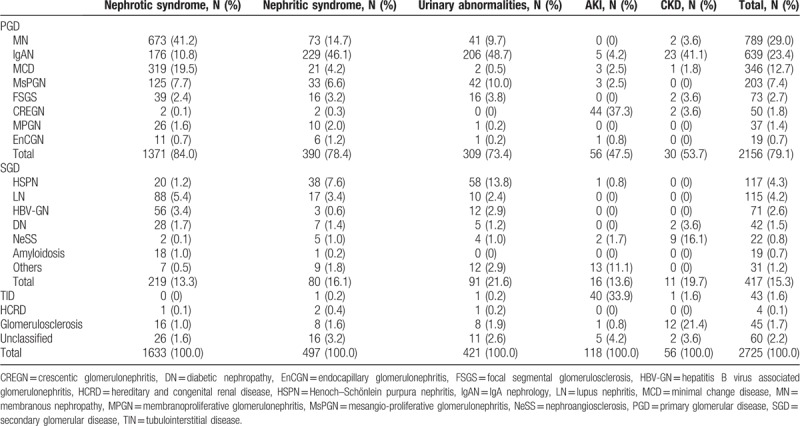
Clinicopathological findings of renal biopsy based on different clinical indications.

As summarized in Table 3, among the patients presenting with nephrotic syndrome, MN was the most common pathological finding, followed by MCD, IgAN, and LN. Among the patients presenting with nephritic syndrome, IgAN was the most common pathological finding, followed by MN, MsPGN, and HSPN. Among the patients presenting with UAB, the most frequent pathological finding was IgAN, followed by MsPGN, MN, and HSPN. The majority cases of AKI were found to have CREGN and TIN. In contrast, IgAN was the most common cause of those presenting with CKD, followed by glomerulosclerosis and NeSS.

We further divided the study duration into 2 periods, 2007 to 2011 (period 1) and 2012 to 2016 (period 2). We then analyzed the spectrum of pathological findings according to different periods. As summarized in Table 4, MN (*P* < .01) and MCD (*P* < .01) were the most prominent subtypes that showed significantly increased prevalence over time, while the prevalence of FSGS (*P* < .01) and non-IgA MsPGN (*P* < .01) decreased significantly over time. There was no significant difference in the prevalence of CREGN, MPGN, and EnCGN between the 2 study periods. The prevalence of HSPN (*P* < .05), LN (*P* < .01), and HBV-GN (*P* < .01) decreased over time, while the prevalence of NeSS (*P* < .01) increased significantly. Finally, the prevalence of TID (*P* < .01) increased significantly, while the prevalence of glomerulosclerosis (*P* < .01) and unclassified findings (*P* < .01) decreased over time.

**Table 4:**
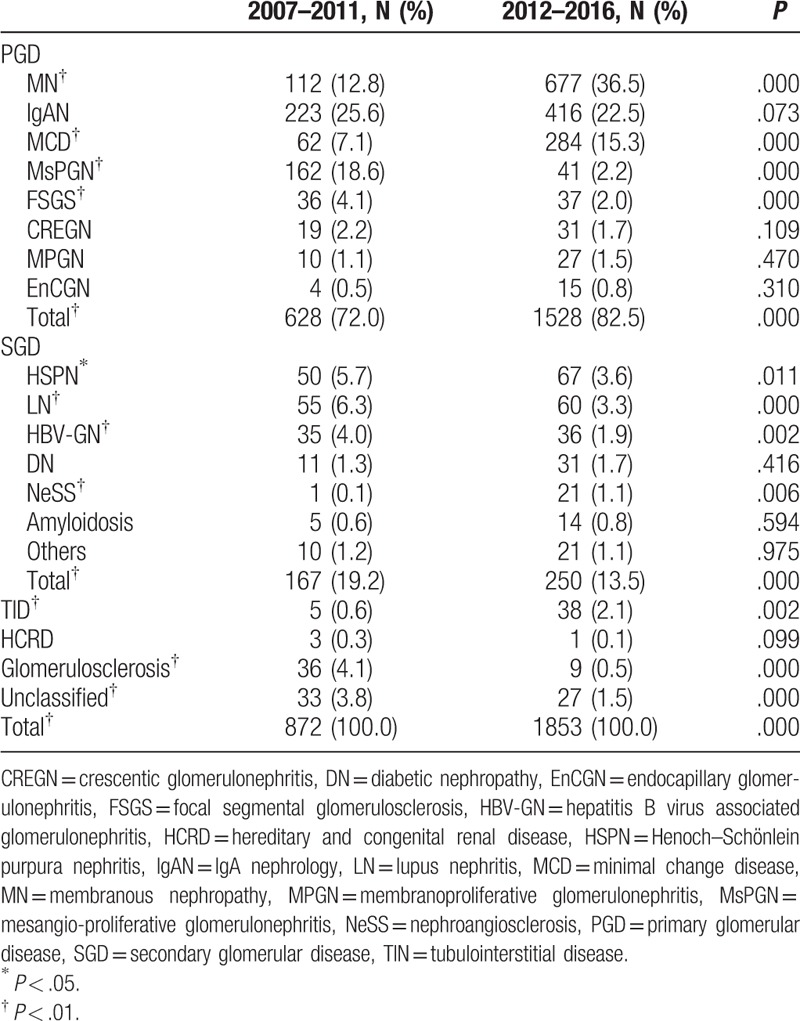
The distribution of pathological findings based on different study periods.

## 5 Discussion

In this study, we found that the most common indication for renal biopsy is nephrotic syndrome, compatible with results from most studies^[3,11,22]^ but not with those from Japan,^[4]^ where nephritic syndrome is the main indication, and with those from Italy,^[17]^ where urinary abnormalities are the main indication. This may result from the geographical variations in criteria for performing renal biopsies and in diagnostic criteria for various renal manifestations. It is still highly debated whether to collect a renal biopsy from patients with isolated hematuria with or without minor proteinuria. It is also likely that patients may be diagnosed with nephritic syndrome in some centers but have urinary abnormalities in others due to different diagnostic criteria, potentially influencing the distribution of clinical indications. This is particularly relevant when the indication for renal biopsy is not nephrotic syndrome. However, most studies did not provide the diagnostic criteria for different renal manifestations. Consequently, a definite conclusion cannot be made in this regard.

**Figure 1:**
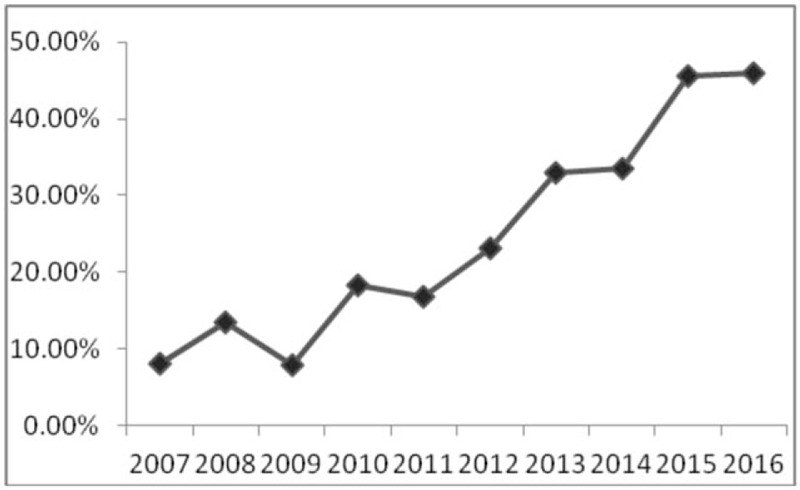
The percentage of MN among all renal biopsies between 2007 and 2016 (*P* < .01).

**Figure 2:**
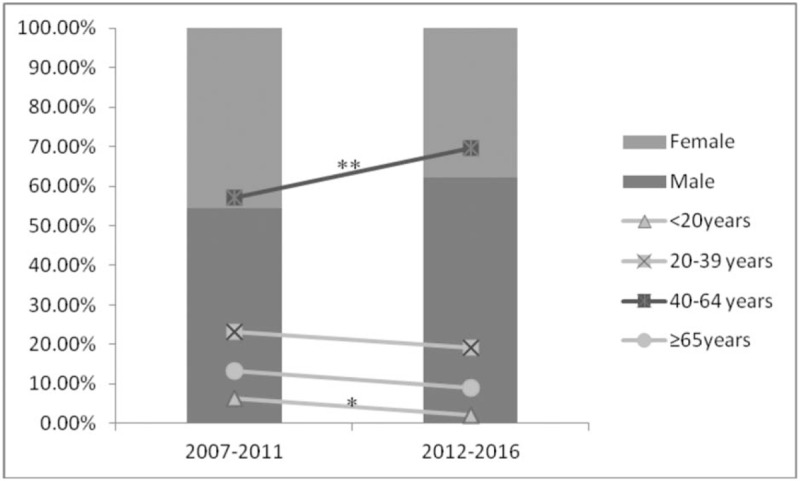
Gender difference (*P* *>* .05) and age differences (^∗^*P* < .05, ^†^*P* < .01) in MN in the different time periods studied.

MN was the most frequent pathological finding in this study, which was first reported in Asian countries; however, other studies have indicated that IgAN^[4–6,19,21]^ or MsPGN (in India)^[23]^ was the most common finding of renal biopsies. Furthermore, a 5.7-fold increase in the proportion of MN from all biopsies was observed between 2007 and 2016 (from 8% to 45.9%, Fig. 1). A previous Chinese study^[12]^ reported that the frequency of idiopathic MN among all patients with PGD doubled from 2003 to 2012, and MN has been identified as the most common origin of adult nephrotic syndrome in many published studies.^[11,17,19,22,24]^ Our study is the first to show that MN is also the most common diagnosis in a renal biopsy registry. Plausible reasons for this finding may include a change in socioeconomic status and environment. Xu et al^[6]^ reported that, among the data from 938 hospitals spanning 282 cities in China from 2004 to 2014, the frequency of MN increased 13% per year, while the proportions of other major glomerulopathy remained stable. They also found that long-term exposure to high levels of particulate matter 2.5 (PM 2.5) was associated with an increased risk of MN, another potential reason for this epidemiologic change. The particulate matter metric refers to the amount of such particles per cubic meter of air; the higher this value, the more serious the air pollution. Although a tendency toward male predominance was observed in the total MN population and this trend increased during the 2 periods, the gender-difference was not significant (Fig. 2). The increase in the proportion of patients with MN was mainly observed in patients in the 40 to 64-year-old age group, as shown in Fig. 2.

FSGS is the most frequent type in American^[25]^ and in Arab in the middle east,^[26]^ and is 1 of the 2 major causes of nephrotic syndrome in children.^[27]^ But it was not common in this study, and the prevalence was decreasing. In the central region of China, the frequency was 3.63%,^[20]^ and in east China was 2.5% of PGD,^[28]^ and in Korea was 5.6%.^[5]^ The reason of the low prevalence was unclear. The frequency of the pathological phenotype was quite different due to geographical locations, races, ages, gender (i.e., LN) and it will change with time due to the changing environment and developing treatment methods, which may explain the low prevalence of FSGS in this region.

HSPN was the most frequent type of SGD in this study. This differs from previous reports, which indicated that the most frequent SGD is LN.^[3,11]^ We propose several reasons for this difference. First, Asians are more susceptible to the development of HSPN than patients of other ethnicities. A study conducted by Gardner-Medwin et al^[29]^ showed that HSPN is more frequent in Asian children than in those that are Caucasian or of African-descent. Second, patients with HSPN often undergo renal biopsy for the adjustment of the immunosuppressive therapy regimen, while patients with LN sometimes start immunosuppressive therapy based on other indications without the assistance of renal biopsy. Third, this study involved teenagers (aged 14–18 years). This population of teenage population had a higher incidence of HSPN (21/97) but a lower incidence of LN (8/97) than observed in adult patients.

The pathological findings of HBV-GN mainly included MN (69/71) and MPGN (2/71). The proportion of patients with HBV-GN was lower in period 2 than in period 1 (from 4.0% to 1.9%). We propose several reasons to explain this change. First, the HBV vaccination program of the Chinese government commenced in 1992 and this led to a decrease in the prevalence of HBsAg in 2006 (from 9.75% to 7.18%), and a concomitant decrease in the prevalence of HBsAg in children below 5 years of age (from 9.67% to 0.96%). This achievement has dramatically changed the epidemiology of HBV infection in China, from a highly endemic status to a moderately endemic one.^[30]^ Second, the development of new antiviral drugs in the past decade has facilitated the control of HBV infection, leading to a decrease in the rate of HBV-related mortality, including HBV-GN.^[31]^

**Figure 3:**
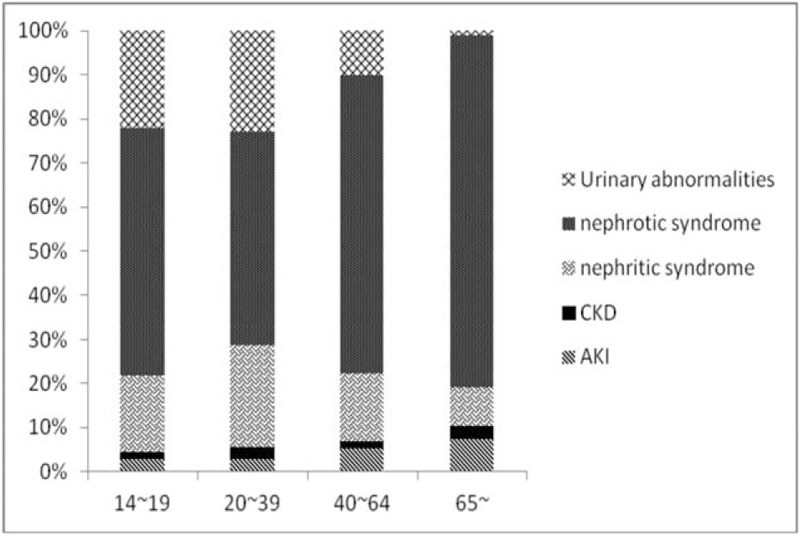
Distribution of different clinical indications in each age group (*P* < .01).

Renal pathologic findings differ between the elderly (≥65 years old) and young patients.^[2,32,33]^ In this study, the elderly constituted 6.3% of the entire cohort. The main indication for renal biopsy of elderly patients in this study was nephrotic syndrome (Fig. 3) and the predominant pathological finding was MN, followed by MCD (Table 1). These results are similar to those of other studies.^[22,32–34]^ However, in a Spanish study, AKI was the most common clinical indication for renal biopsy in elderly patients,^[35]^ with the leading pathology being amyloidosis. The rising incidence of nephroangiosclerosis (Table 4) may reflect an increase in the age of patients undergoing renal biopsy. However, further studies should be performed for clarification.

There are still several limitations in this study. As the time period is only 10 years, it is better to conduct a longer duration study to reveal the time prevalence of the renal biopsy. Besides, the investigation of clinical data in this study is brief, which can be further studied in the future.

## 6 Conclusion

This is the first study to describe the epidemiology of renal biopsy findings from northeast China. MN was the most common pathological finding, with a significant increase in the prevalence observed in recent years, while HSPN and LN were the most common SGDs. Our findings provide regional data regarding renal pathology and serve as the basis for further research.

## Author contributions

**Conceptualization:** Sensen Su.

**Data curation:** Sensen Su, Jinyu Yu, Yu Wang, Zhonggao Xu.

**Formal analysis:** Sensen Su, Zhonggao Xu.

**Investigation:** Sensen Su, Jinyu Yu, Yue Wang, Jia Li, Zhonggao Xu.

**Methodology:** Jinyu Yu, Zhonggao Xu.

**Project administration:** Sensen Su, Zhonggao Xu.

**Software:** Sensen Su.

**Supervision:** Zhonggao Xu.

**Validation:** Sensen Su, Zhonggao Xu.

**Visualization:** Zhonggao Xu.

**Writing – original draft:** Sensen Su, Jinyu Yu, Yue Wang.

**Writing – review & editing:** Sensen Su, Yue Wang, Yu Wang, Jia Li, Zhonggao Xu.

Zhonggao Xu orcid: 0000-0003-4302-4136.
